# Altitudinal Barrier to the Spread of an Invasive Species: Could the Pyrenean Chain Slow the Natural Spread of the Pinewood Nematode?

**DOI:** 10.1371/journal.pone.0134126

**Published:** 2015-07-29

**Authors:** Julien Haran, Alain Roques, Alexis Bernard, Christelle Robinet, Géraldine Roux

**Affiliations:** 1 INRA, UR633 Zoologie Forestière, F-45075 Orléans, France; 2 Université d’Orléans, Orléans, France; Instituto de Higiene e Medicina Tropical, PORTUGAL

## Abstract

Mountain ranges may delimit the distribution of native species as well as constitute potential barriers to the spread of invasive species. The invasive pinewood nematode, *Bursaphelenchus xylophilus*, is a severe forest pest inducing pine wilt disease. It is vectored in Europe by a native long-horned beetle, *Monochamus galloprovincialis*. This study explored the potential of the Pyrenean chain to slow or prevent the natural spread of nematode-infested beetles from the Iberian Peninsula, where the nematode is established and is expanding its range, towards France and the rest of Europe. An analysis of the genetic structure and migration patterns of the beetle populations throughout the Pyrenean mountain range was combined with a spread model simulating the potential movements of nematode-infested beetles across it. The central part of the Pyrenees, which corresponds to the highest elevation zone, was shown to prevent gene flow between the French and Spanish populations of *M*. *galloprovincialis* on each side of the mountains. Conversely, strong admixture was detected between populations located on both sides of low elevation hills, and especially at the east and west extremities of the mountain range. Simulations of the spread of nematode-infested beetles under various thresholds of beetle survival and pine wilt disease expression gave results consistent with the variation in genetic make-up, suggesting that western and eastern hillsides may represent corridors favoring natural spread of the nematode from the Iberian Peninsula to France. Simulations also showed that temperature rise due to climate change may significantly reduce the extent of the barrier formed by highest elevations. Our results support the hypothesis that the Pyrenean chain represents a partial barrier to the natural spread of nematode-infested beetles. These results, which have to be considered together with potential human-assisted long-distance spread of the nematode, highlight priority zones for future pest monitoring and management programs. More generally, such an integrated approach could be used to assess the role of mountain chains in the potential spread of other invasive pests.

## Introduction

Introduction of species beyond their native ranges has increased dramatically over recent decades due to intensification of international trade [1, 2, 3]. When successfully established in new areas, introduced species may expand their ranges and can cause major environmental disturbances together with significant economic losses [4]. The local range expansion of introduced organisms depends on their dispersal ability, which is a critical parameter for development of containment measures [5]. In most cases, this expansion occurs in heterogeneous environments, where the spatial and temporal distributions of biotic and abiotic constraints vary [6]. This heterogeneity determines the influence and scale of landscape effects on dispersal of species [7]. Depending on biological and physical constraints affecting the dispersing species, the spatial distribution of these constraints may constitute negative barriers or positive corridors determining range expansion of the invading species. Identifying environmental factors underlying the efficiency of dispersal of invasive species is essential in developing suitable management measures. Mountainous areas are major components of landscape heterogeneity, exhibiting contrasting climatic conditions that have historically shaped the genetic structures of species by affecting connectivity of landscapes [8, 9, 10]. The Pyrenean chain is a major mountain range in South-Western Europe. Due to its high elevation (up to 3404 m) and its spatial extent (more than 400 km long) this chain is an altitudinal obstacle between the Iberian Peninsula and the rest of Europe that has strongly affected the distributions and genetic structures of native species. Phylogeographic studies have highlighted the role of this barrier in shaping intraspecific lineages [8] and as a major contact zone for post glacial range expansion in European biota [11]. Owing to the effect of this barrier on the dispersal of native organisms, the Pyrenean chain could therefore play an important role in slowing the spread of invasive species from the Iberian Peninsula to the rest of Europe or conversely.

The pinewood nematode (PWN), *Bursaphelenchus xylophilus* (Steiner & Burher, 1934) Nickle, 1970 (Nematoda, Aphelenchoididae) is the causal agent of the pine wilt disease (PWD). Under suitable climatic conditions, this pest is able to kill susceptible pine trees within a few months [12]. Though it causes limited damage in its native range in North America [13], it has resulted in massive mortality to native pine forests [14] in its area of introduction in East Asia (Japan-1905, China-1982, Taiwan-1985, Korea-1988). PWN was detected for the first time in Europe in Portugal in 1999, in the peninsula of Setubal [15]. Despite intensive containment measures, it has quickly expanded its range through most of Portugal and has entered Spain, where it is under eradication [16]. The natural dispersal of the PWN is exclusively done through the activities of longhorned beetles in the genus *Monochamus* (Coleoptera, Cerambycidae) [17]. So far, the widely distributed *Monochamus galloprovincialis* is the only known PWN vector in Europe [18]. *M*. *galloprovincialis* females oviposit in stressed or freshly dead pine trees, after which the larvae develop and pupate within the wood [19]. The nematodes migrate to the pupal chamber and moult to a specific larval stage that enters the tracheae of callow adult *Monochamus* prior to their emergence. Transmission of PWN takes place either during maturation feeding in the crowns of living pines (primary transmission) and also during oviposition on declining trees (secondary transmission) [17, 20, 21]. In both cases, individuals carrying the nematode (here after called PWN-infested beetles) can fly and disperse the nematode. Consequently, the natural spread (i.e. non-human mediated) of PWN in Europe depends mainly on the dispersal abilities of the vector beetle.

Little is known on the effective dispersal of *M*. *galloprovincialis* in the field and how environmental parameters affect this dispersal. Based on flight mill experiments, the physiological flight capacity of this beetle was estimated to be an average of 16 km over the lifespan of the beetle [22] whereas mark-release-recapture experiments conducted in Spain revealed that adult beetles may disperse up to 22 km in the field [23]. These methods are known to overestimate and underestimate dispersal abilities respectively [22, 24]. In addition, mountain chains are complex environments where variations in topography, pine densities, pine species and temperatures are likely to play a significant role in dispersal of this beetle, and may act as a disrupting factor. To date, no data are available on environmental conditions influencing dispersal of *M*. *galloprovincialis*.

Microsatellite markers are broadly used to infer population genetic structure [25], gene flow between populations [26], individual migration events [27, 28] and to assess the effects of landscape on dispersal [7]. Estimates of the genetic structure of *M*. *galloprovincialis* populations across the Pyrenees can thus provide valuable information on the location of either barriers or corridors to dispersal of this species, and therefore act as determinants of the range expansion of PWN. However, for native species such as *M*. *galloprovincialis*, the observed genetic structure also results from past demographic processes whose time scales may be difficult to determine. Consequently, it is challenging to infer precisely the current dispersal abilities of the vector from the genetic structure of the sampled populations [29]. To test dispersal in current environmental conditions, model-based simulations represent a consistent and complementary approach. Spread models are widely used to describe and simulate potential dispersal of invasive species [30, 31, 32, 33]. Although based on the current known biology of the species, models allow the testing of several scenarios under current and future environmental conditions. Such spread models have been used to predict the spread of the PWN and associated PWD in Japan [34, 35, 36] and China [37]. In Europe, a large-scale spread model was applied to identify the most likely factors determining spread of PWN, taking into account short-distance flight as well as human-mediated longer-distance transportation of infested individuals of *M*. *galloprovincialis*. This model assumes that the spread potential in Europe is similar to that already observed in China [38]. To date, no study has focused on the potential spread of PWN-infested populations at local scale, in an environment exhibiting strong landscape heterogeneity.

Dispersal of PWN results from a combination of natural vector-mediated spread and human long-distance transportations. Given the progression of the PWN in the Iberian Peninsula [39] and the possible economic consequences for European countries [40], it is crucial to consider both quarantine policy and potential barriers to the natural spread of this alien species in order to define suitable management measures. The aim of the present study is to focus on the potential natural dispersal of PWN through the Pyrenean chain, which is a strategic boundary between the infested area (Iberian Peninsula) and the rest of Europe. Environmental and physical conditions associated with this mountain range can potentially affect the dispersal of *M*. *galloprovincialis* and the spread of PWN. We address the following questions: 1) Does the Pyrenean chain represents a barrier to natural dispersal of *M*. *galloprovincialis*? 2) Could this chain slow or stop the natural spread of PWN under current and future climate conditions? To answer these questions, we combined a population genetic analysis and a modeling approach, using microsatellite markers to elucidate the genetic structure and migration patterns of *M*. *galloprovincialis* and a spread model to simulate the invasion of PWN-infested beetles across this mountain range.

## Materials and Methods

### Field sampling

**Fig 1:**
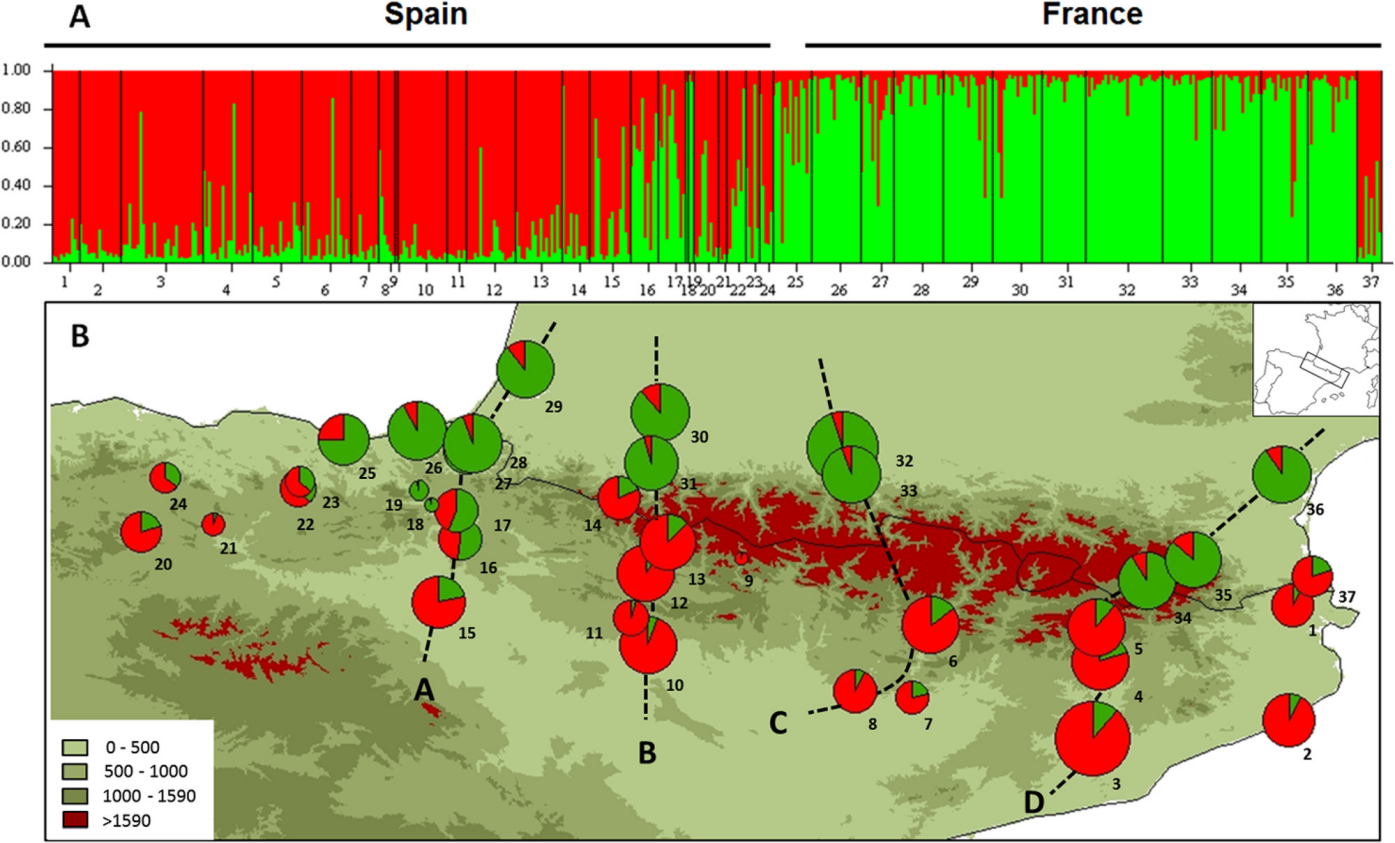
Bayesian clustering of individuals and populations of *M*. *galloprovincialis*. A) Barplots of individual assignment of the 485 individuals for K = 2. B) Membership of populations (sum of membership of individuals). Background refers to elevation.

The sampling sites were selected in order to cover both sides of the entire Pyrenean chain. They included 9 sites on the Northern side (France) of the Pyrenees and 13 on the Southern side (Spain), forming 4 North-South transects (Fig 1). To detect potential effects of environmental factors (temperature, host tree species and elevation) on genetic differentiation and gene flow of *M*. *galloprovincialis*, these sampling sites were located along a gradient of elevation on each side of the Pyrenean chain. Transects were located close to the main road axes crossing the Pyrenees, as they are mostly situated in valleys. These areas thus represent potential corridors for the dispersal of this beetle through natural or human mediated dispersal (S1 Fig). Additional sites were sampled on the Western side of the chain (populations 25 and 26) and along the Mediterranean Sea (populations 1 and 37) to estimate gene flow along the low elevation coastal areas. In order to increase the coverage of the sampling, several additional populations and isolated individuals were included in this study (2, 9, 18–24). Specimens were trapped at all locations on private land with permission of the land owners. No more specific permissions were required and these field studies did not involve endangered or protected species. Specimens were captured using multifunnel traps and a specific volatile attractant (Galloprotect, SEDQ, Spain). A set of 3 traps was installed on each site from late June to the end of October and specimens were collected every 3 weeks. After identification to species level, specimens were stored in 99.5° ethanol at 4°C. *M*. *galloprovincialis* was recorded at all sites except in Vielha (Table 1). Despite four months of trapping, this species was difficult to capture in 3 localities above an altitude of 1400 m (Font-Romeu, Col de la Pierre Saint Martin and Canfranc). Overall, we obtained 26 samples (430 specimens) supplemented by specimens from 11 sites collected previously, giving a total number of 37 sampling sites and 485 specimens to be genotyped.

**Table 1:** Details of aggregates of *M*. *galloprovincialis* considered across the Pyrenees.

Code	Location	Country	Transect	Year	Long.	Lat.	Elevation (m)	*Pinus*	N	A. rich.	P.	Ho	He	Fis
1	Capmany	Spain	_	2014	2.95	42.36	100	*P*. *halepensis*	10	3.03	0.02	0.483	0.586	0.155
2	Tossa de Mar	Spain	_	2012	2.93	41.72	21		15	3.04	0	0.455	0.496	0.153
3	Castellbell	Spain	D	2013	1.84	41.62	190	*P*. *halepensis*	30	3.42	0.06	0.493	0.574	0.146
4	Olvan	Spain	D	2013	1.88	42.05	520	*P*. *pinea/nigra*	18	3.25	0.04	0.486	0.481	-0.015
5	Baga	Spain	D	2013	1.86	42.24	794	*P*. *sylvestris*	18	3.29	0.08	0.474	0.52	0.104
6	La Pobla de S.	Spain	C	2013	0.94	42.25	646	*P*. *halepensis*	18	3.55	0.19	0.501	0.568	0.139
7	Camarassa	Spain	_	2013	0.84	41.85	271	*P*. *halepensis*	6	_	_	_	_	_
8	Castillonroy	Spain	C	2013	0.52	41.88	402		10	3.31	0.03	0.439	0.494	0.101
9	Torla	Spain	_	2012	-0.11	42.62	1197		1	_	_	_	_	_
10	Huesca 1	Spain	_	2013	-0.63	42.14	419	*P*. *pinea*	7	_	_	_	_	_
11	Huesca 2	Spain	B	2013	-0.72	42.29	552	*P*. *halep*.*/sylv*.	18	3.45	0.04	0.437	0.539	0.169
12	Jaca	Spain	B	2013	-0.64	42.54	870	*P*. *sylvestris*	18	3.39	0.04	0.457	0.532	0.125
13	Canfranc	Spain	B	2013	-0.52	42.71	1502	*P*. *nigra*	17	3.72	0.13	0.525	0.581	0.108
14	Col St-Martin	Spain	B	2013	-0.79	42.96	1590	*P*. *uncinata*	10	3.45	0.12	0.500	0.524	0.027
15	Falces	Spain	A	2012	-1.79	42.38	360		15	3.48	0.21	0.458	0.517	0.131
16	Beriain	Spain	A	2012	-1.67	42.73	613		10	2.51	0	0.408	0.449	0.106
17	Marcalain	Spain	A	2012	-1.69	42.89	550		10	3.13	0.07	0.430	0.494	0.088
18	Iruzun	Spain	_	2012	-1.83	42.92	553		1	_	_	_	_	_
19	Lekunberri	Spain	_	2012	-1.9	43,00	507		2	_	_	_	_	_
20	Ona	Spain	_	2012	-3.44	42.77	1055		9	_	_	_	_	_
21	Espejo	Spain	_	2012	-3.04	42.81	715		3	_	_	_	_	_
22	Vitoria	Spain	_	2012	-2.57	43.01	760	*P*. *nigra*	7	_	_	_	_	_
23	Aramayona	Spain	_	2012	-2.56	43.05	627		5	_	_	_	_	_
24	Villanueva de M.	Spain	_	2012	-3.31	43.07	452		5	_	_	_	_	_
25	Itziar	Spain	_	2013	-2.32	43.28	200	*P*. *nigra*	14	2.92	0.01	0.411	0.463	0.094
26	Irun	Spain	_	2013	-1.91	43.33	23	*P*. *pinaster*	18	2.95	0	0.399	0.454	0.114
27	Etchalar	Spain	A	2013	-1.63	43.22	273	*P*. *radiata*	12	2.8	0	0.298	0.447	0.286
28	Sare	France	A	2013	-1.6	43.26	303	*P*. *nigra*	18	2.74	0	0.370	0.449	0.184
29	St-Vincent	France	A	2013	-1.31	43.67	32	*P*. *pinaster*	18	2.89	0	0.370	0.435	0.092
30	Orthez	France	B	2013	-0.56	43.43	180	*P*. *nigra*	18	2.88	0	0.300	0.424	0.303
31	Oloron	France	B	2013	-0.61	43.15	302	*P*. *strobus*	16	2.76	0.03	0.368	0.417	0.091
32	Vieuzos	France	C	2013	0.45	43.24	449	*P*. *nigra laricio*	28	2.7	0.03	0.345	0.428	0.198
33	Garros	France	C	2013	0.5	43.09	560	*P*. *nigra laricio*	18	2.79	0.03	0.306	0.391	0.145
34	Font-Romeu	France	D	2013	2.14	42.5	1432	*P*. *sylvestris*	18	2.83	0.02	0.415	0.474	0.138
35	Prades	France	D	2013	2.4	42.61	410	*P*. *halepensis*	17	2.87	0.01	0.400	0.460	0.149
36	Font froide	France	D	2013	2.89	43.09	235	*P*. *pinaster*	18	2.75	0.01	0.317	0.421	0.219
37	Argelès	France	_	2013	3.06	42.52	45	*P*. *halepensis*	9	_	_	_	_	_
_	Vielha	Spain	_	2013	0.82	42.69	1280	*P*. *sylvestris*	0	_	_	_	_	_
Mean South side		Spain								3.329	0.084	0.467	0.522	0.102
(Mean±SD)										0.299	0.065	0.034	0.039	0.051
Mean North side		France								2.823	0.011	0.358	0.438	0.167
(Mean±SD)										0.07	0.01	0.044	0.023	0.072

Code: identification number of aggregates (see Fig 1); Long./Lat., geographic coordinates; *Pinus*, dominant host tree species in sampling site; N, population size; A. rich., mean allelic richness over loci calculated under rarefaction; P., average private allelic richness over loci; He and Ho, expected and observed heterozygosity; *F*_is_, multilocus *F*_is_ estimate; “_”, aggregate not considered as a population for genetic analysis.

### DNA extraction and microsatellite analysis

Genomic DNA was extracted from two legs per individuals using a Nucleospin Kit (Macherey–Nagel, Düren, Germany), following manufacturer’s instructions. Each DNA extract was equilibrated with sterile water to a concentration ranging from 10 to 30 ng/μL. Individuals were genotyped at 12 loci previously described (Mon01, Mon08, Mon17, Mon23, Mon27, Mon30, Mon31, Mon36, Mon41, Mon42 & Mon44) [41]. Multiplexed PCR were performed in a 10 μL reaction volume using 1 μL of genomic DNA, 0.4 U of DreamTaq DNA Polymerase (Thermo Scientific), 0.75 μL Dream Taq Green Buffer (including 20 mM MgCl_2_, Thermo Scientific), 1 μM Betaine, 0.24 μL dNTP (10 μM) and deionized H_2_O. PCR amplifications were run on a Veriti 96 well fast Thermal cycler (Applied biosystems) using the following settings: a first denaturation step at 95°C over 10 min; 40 cycles of denaturation (30 s at 95°C), hybridization (30 s at 55°C) and elongation (1 min at 72°C); and a final elongation step at 72°C over 10 min. One μL of PCR product was denatured within a mix of 10 μL of formamide and 0.3 μL of 600 Liz marker before being run on an ABI PRISM 3500 sequencer (Life Technologies). Genotypes were read using the software GeneMapper V 4.1 (Applied Biosystems). For primer sequences and multiplexe details please refer to Haran & Roux [41].

Basic genetic parameters (Heterozygote frequencies: He and Ho) and *F*_is_ were calculated using Genalex 6.5 [42]. The mean allelic richness and private allelic richness of each population was computed by regression using HP-RARE [43], to correct bias involved by unequal sampling size. Deviation from HWE at each locus was tested using Genepop 4.2 [44], with a False Discovery Rate correction [45]. We also used Genepop 4.2 [44] to test the presence of linkage disequilibrium between loci. The frequency of null alleles at each locus and for each population was estimated using FreeNA [46]. Signatures for recent demographic events such as bottlenecks among populations were assessed using the program Bottleneck [47]. Deviations from mutation drift equilibrium implemented in this program were computed using the Stepwise Mutation Model (SMM, 95%) and the Two-Phase Model (TPM, 5%). Significance of deviations were tested using the Wilcoxon test, following recommendations of authors for microsatellite analysis [47].

### Population differentiation and clustering

Population differentiation was estimated using the unbiased Pairwise *F*_st_ implemented in the program FreeNA [46]. We used the *ENA* method to correct the potential positive bias induced by the occurrence of null alleles on *F*_st_ estimation. Tests for population differentiation significance were made using FSTAT v. 2.9.3.2 [48] based on 325,000 permutations (level for significant differentiation set to p<0.0001). The estimation of unbiased genetic distance based on allele frequency requires a minimum population size. An approximate number of 20 individuals per population is usually advised for structured populations (Fst>0.05) [43]. Due to sampling difficulties, nine scattered populations were represented by a smaller number of individuals (N<16). These populations were included in analysis for their contribution to the spatial coverage, bearing in mind that population genetic estimates of differentiation have to be carefully considered in these cases. Isolation by distance between populations was tested through a Mantel test implemented in Genepop 4.2 [44]. Correlations were computed between linear geographic distances and pairwise genetic differentiation (*F*_st_ /1- *F*_st_) for 10,000 permutations. Isolation by distance was assessed on the whole dataset and within each side of the mountain range.

The genetic clustering of populations was computed using the Bayesian approach provided by the software STRUCTURE 2.3.4 [28]. STRUCTURE attributes to each individual a likelihood of membership to a predefined number of clusters, minimizing deviation from linkage DIS-equilibrium between loci. Analyses were performed using the “correlated allele frequencies” without “sampling populations as prior”. We used the “admixture model” due to the relatively small scale of the study and because *M*. *galloprovincialis* has good flight performances [22], reaching up to 22.1 km per year [23]. The “optimal” number of clusters (K) was determined using the Delta-K method [49]. The genotypes were analyzed by running 10 repeats of a 100,000 burn-in period followed by 500,000 replicates of Markov Chain Monte Carlo (MCMC), for K values ranging from 1 to 10. We used the online service STRUCTURE HARVESTER [50] to visualize the evolution of Delta-K (mean likelihood of K/ variance of likelihood for the same K) through the values of K tested. The value of K maximizing likelihood and minimizing variance was chosen as optimal for this dataset. Among the 10 repeats, the one showing the highest mean value of Ln likelihood was selected for graphical representation. For control purposes, clustering analysis was performed with and without loci exhibiting significative null allele frequency. We used ArcGis 9.3 (ESRI) to plot the membership of individuals (Qvalue) to the predefined clusters for each population.

### Detection of migration

We estimated the proportion of admixture along the four transects to detect the signature of migration events across the Pyrenean chain. Admixture in populations was computed using the coalescent-based maximum Likelihood method implemented in the program LEA [51]. This method was chosen due to its broad applicability and its very low bias for ancient hybridization [52]. LEA estimates the likelihood of a hybrid population to belong to two predefined parental populations. Along each transect, we defined parental populations at the two extremities. Admixture was calculated for each population between these parental populations, based on 10 computations of 11,000 iterations.

The frequency and origin of first generation migrants was estimated using assignment tests implemented in GeneClass 2.0 [53]. We used the Bayesian method [54] to test the likelihood of each individual to belong to any of the 26 populations including the one of origin (Likelihood (L) = L population of origin / L maximum in all populations). Calculations were performed based on the Monte Carlo resampling method [55], using 10,000 simulated genotypes, with a threshold for migrants detection set to 0.001. For such analysis, we corrected null alleles in the dataset to minimize the risk of bias in the indices. The loci significantly affected (>10%) were corrected using the INA method [46] assuming a single null allele state shared by all populations.

### Modeling the potential spread of infested populations of *M*. *galloprovincialis* across the Pyrenees

We used a mathematical model to explore the effect of physical and biological parameters on the spread of a hypothetical infested population of *M*. *galloprovincialis* across the Pyrenees (from Spain to France). We modeled the spread of an endemic vector species (*Monochamus sp*.) carrying an invasive organism (PWN) and able to transmit this invasive species to resident vector populations. In this case study, each partner of this PWN-beetle association is affected by the dynamics of the other. The PWN needs the vector to disperse and the vector benefits from the mortality of trees caused by the PWN to find more suitable resources and reproduce. Thus, the model has to consider both sides of the association: the pre-existence of natural population of *M*. *galloprovincialis* in the area of study (resident populations) and the effect of PWN outbreaks on the densities of this species. The potential spread of PWN and its damage at relatively local scale (i.e., non-human mediated dispersal) was described by a reaction-diffusion model [37, 38]. This model simulates both population dispersal and logistic growth:

$$\frac{\partial N}{\partial t}=D\left(\frac{\partial^{2}N}{\partial x^{2}}+\frac{\partial^{2}N}{\partial y^{2}}\right)+f\left(N\right)$$ (1)

$$f\left(N\right)=\varepsilon N\left(1-\frac{N}{K}\right)$$ (2)

where *N* is the population density of *M*. *galloprovincialis*, *x* and *y* the geographic coordinates (in km), and *t* the time (i.e., the year as the vector has one generation per year in the geographic area considered in this study). *D* is the diffusion coefficient (km^2^/year). We used the value estimated in China for *Monochamus alternatus*, *D* = 6.48 km^2^/year [37], due to the lack of accurate data to estimate this parameter more precisely in Europe. *f* is a logistic growth function with *ε* the growth rate and *K* the carrying capacity of the local environment. The growth rate, *ε* = 2.17, was estimated based on the ratio of host trees killed by PWN in China [37]. As there are insufficient data to estimate this parameter in Europe, we employed this estimate in our model. Values of *D* and *ε* were also used in a previous spread model applied to PWN in Europe [38]. The carrying capacity, *K*, is the maximum population density that the local environment can maintain. *K* depends on the number of declining host trees available for the development of *M*. *galloprovincialis*. This parameter depends whether the PWN is present or not.

Here, we explicitly describe the symbiotic relationship between the PWN and its vector considering that the carrying capacity changes at the PWN arrival. We assume that *K* = *K*_0_ before and *K* = *K*_*i*_ after PWN arrival, with a ratio between *K*_i_ and *K*_0_ estimated from trapping PWN-infested and PWN-free areas in Portugal. The parameter *K*_0_ was estimated using results of trapping in the Pyrenees (where the PWN is not yet present) and pine tree density nearby. In addition, we assume that the density of the resident population of the vector before the arrival of PWN nearly reaches local carrying capacity (*R* = α *K*_0_, with α = 0.9). The pine density was estimated based on the percentage of land covered by pine species (data provided by EFI, http://www.efi.int/; [56], assuming that 100% cover represents 156000 trees/km^2^ (approximately the highest pine density in maritime pine forests). Given the wide range of hosts for *M*. *galloprovincialis*, we did not differentiate pine species and considered overall pine density when calculating the carrying capacities and resident population densities [19, 57].

To account for the invasion dynamics of infested populations (*N*) and the recruitment of resident populations (*R*), we refine the growth function (2) according to the invasion stage. More precisely, we consider the following four chronological steps, assuming that PWN arrives in year *t* = *n* at a given location:

1. Before the PWN arrival (*t*<*n*), absence of infested vectors:
*N* = 0 and *K* = *K*_0_
2. PWN introduction at *t* = *n*, a certain amount of trees are weakened or killed by the PWN, the carrying capacity increases from *K*_0_ to *K*_*i*_. But at this stage, there is not yet recruitment of resident populations as newly infested beetles will emerge the year after:
  $$f\left(N\right)=\varepsilon N\left(1-\frac{N+R}{K_{i}}\right)$$ (3)
3. At *t* = *n*+1, offspring of both infested and resident populations carries the PWN when emerging from PWN-infested trees. Thus, we consider the recruitment of resident populations:
  $$f\left(N\right)=\varepsilon \left(N+R\right)\left(1-\frac{N+R}{K_{i}}\right)$$ (4)
4. In the following years (t>n+1), the whole population is infested (no further recruitment):
  $$f\left(N\right)=\varepsilon N\left(1-\frac{N}{K_{i}}\right)$$ (5)

With this schematic approach, the aim is to account for the main processes and provide a preliminary analysis of the effects of the Pyrenean chain on spread of infested beetles rather than giving an accurate prediction of the spread rate. Since direct validation was impossible (because PWN is not yet present in the Pyrenees), an indirect validation of the vector spread model was done by comparing simulations to the results of the genetic study.

### Description of spread simulations

The study area covers the Pyrenean chain and adjacent sides (41.52–44.22° N, -3.24–3.96° E, ≈ 300 km North to South of the ridgeline and ≈ 700 km East to West). Simulations were made over a grid of cells of 1 km x 1 km covering this area, using R language [58]. We simulated the potential spread of PWN-infested populations starting with 200 infested beetles at time *t* = 0 from the South-West of the Pyrenees. Simulations were done until all favorable areas of the grid were colonized. Areas were considered colonized as soon as the population density exceeded 1 infested insect per km^2^. To measure the time needed to cross the Pyrenees from South to North in different points of the mountain range, we simulated the spread along the four transects (A, B, C & D). Simulation started from the Spanish extremity of transects (South of the Pyrenees) and we measured the number of generations required to reach the French extremity (North of the Pyrenees).

### Effects of temperature and elevation

Temperature affects the developmental rate of *M*. *galloprovincialis* [59]. Since this insect has its larval development that spans both summer and winter, it is likely to be affected by winter temperatures encountered at high elevations in the Pyrenees. Winter temperatures have been shown to be good indicator for survival and development of pine associated insects with a winter larval development [60, 61]. We considered the mean minimum temperature in winter (December, January and February) over 1950–2000, called TN hereafter (downloaded from http://www.worldclim.org, [62]). To determine the temperature threshold for survival, we extracted TN from 353 sites where *M*. *galloprovincialis* was recorded throughout Europe. We then obtained the TN value below which this species was not or rarely found. As there is probably a gradual effect of low temperatures and large variability on insect survival instead of a sharp and common threshold, we tested various values (0, -2, -5, -7, and -10°C). The highest elevation at which *M*. *galloprovincialis* can survive and spread is closely linked to temperature. Therefore we also tested the following elevation thresholds: 1200, 1500, 1590, 1700 and 2000 m based on trapping results. In the model simulations, we considered no beetle survival and no PWN transmission below the temperature threshold and above the elevation threshold, respectively.

### Pine Wilt Disease expression and host tree decline

PWN transmission to a host tree does not necessarily involve the expression of pine wilt disease (PWD) and tree decline. PWD expression depends on environmental factors and notably summer temperatures and is generally not observed in areas where the mean temperature in July is below 20°C [63]. Therefore, in this case, there is no additional resource for the vector and the carrying capacity remains the same before and after PWN invasion (*K*_*i*_ = *K*_0_). These areas are clearly unfavorable for the natural expansion of the nematode compared with warmer areas where PWN cause outbreaks of vector density. In another set of simulations, we therefore considered that infested PWN populations can spread only where the mean temperature of July is above 20°C. Since temperature is predicted to increase by 2.8–4.0°C in the Pyrenees over the next century [64], the area where they can spread could be modified. To explore the effects of climate change in the near future, we used putative values of +1 and +2°C in the simulations.

## Results

### Genetic analysis

The number of alleles per locus ranged from 2 to 18, with a mean value of 7.23 over the 26 populations. The average allelic richness of populations over all loci ranged from 2.51 to 3.72 and the average private allelic richness ranged from 0 to 0.21 (Table 1). Both allelic and private allelic richness were significantly higher in populations of the Southern side of the Pyrenean chain. *F*_is_ calculated over populations indicates a slight excess of homozygotes; values ranged from -0.016 to 0.303 for an average value of 0.167 in the Northern side and 0.102 in the South of the mountain range (Table 1). Significant deviations from Hardy Weinberg Equilibrium after FDR correction were detected for the loci Mon27, Mon30 and Mon35. FreeNA identified significant null alleles frequency (>10%) for Mon01 (92%), Mon27 (96%), Mon30 (50%) and Mon35 (38%). No linkage disequilibrium was detected among the 12 loci. Deviation from mutation drift equilibrium tested through Bottleneck showed that only one population exhibited a significant signature of recent bottleneck (Beriain, heterozygosity excess: p = 0.032). Heterozygosity deficit was detected in two populations (Falces and Oloron, heterozygosity deficiency: p = 0.028 and 0.038 respectively).

### Population genetic structure

**Fig 2:**
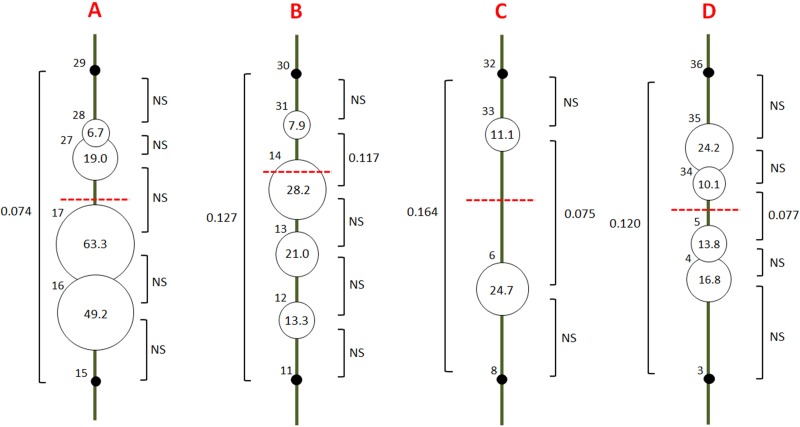
Pairwise *F*_*st*_ and likelihood of hybrid populations along the 4 transects (green line). Numbers on the top left of each circle refer to population code (see Fig 1). Values attached to brackets refer to the unbiased estimates of pairwise *F*_st_ calculated under *ENA* correction (NS = non-significant). The red dotted line shows the placement of the highest ridgeline. Values in the circles and their relative size show the percentage of likelihood to belong to the parent population of the opposite of the ridgeline. Populations used as parents to determine likelihood of hybrid populations are represented as black spots.

Pairwise *F*_st_ computed under the *ENA* correction of the 26 populations was higher than 0.05 for 86% of population pairs, but differentiation was significant (p<0.0001) only for 28.8% of pairs (S1 Table). We observed a ratio of significant pairwise differentiation that was higher between populations of the two sides of the mountain range (72.6%) than between populations of the same side (3.4%). The differentiation was generally moderate to low, the mean pairwise *F*_st_ over the whole dataset was 0.0657 (SD: 0.0423). Mean pairwise *F*_st_ among populations of the same side was 0.0375 (SD: 0.0263), while this distance was 0.0922 (SD: 0.0372) between populations on the two sides. The maximum differentiation (*F*_st_ = 0.185) was observed between Castillonroy (Spain) and Garros (France). Along transects, we did not observe significant pairwise *F*_st_ between populations of the same side (except between Falces and Beriain, Fig 2). Isolation by distance was significant for the whole dataset (p = 0.009), between populations within the Northern side (p = 0.037) and within the Southern side (p = 0.003).

STRUCTURE identified an optimum of two clusters in the dataset through the Delta-K method (ΔK K2 = 1606.68, K3 = 10.70; Fig 1A). We found two distinct genetic clusters with a clear split between populations on each side of the mountain range (Fig 1B). However, some scattered individuals were not assigned to the same cluster within their populations of origin. We did not observe distinct variation in assignment to clusters among the gradients of elevation. Assignment of individuals and populations to one of the clusters was less marked on the Western side of the chain. Analysis using the parameter “No admixture model” gave similar results. Individual assignment (Q values) with and without null alleles did not give strong differences in the patterns observed (mean difference in cluster assignment over all individuals was 2.6%, SD = 3.5%).

### Admixture and migration

**Fig 3:**
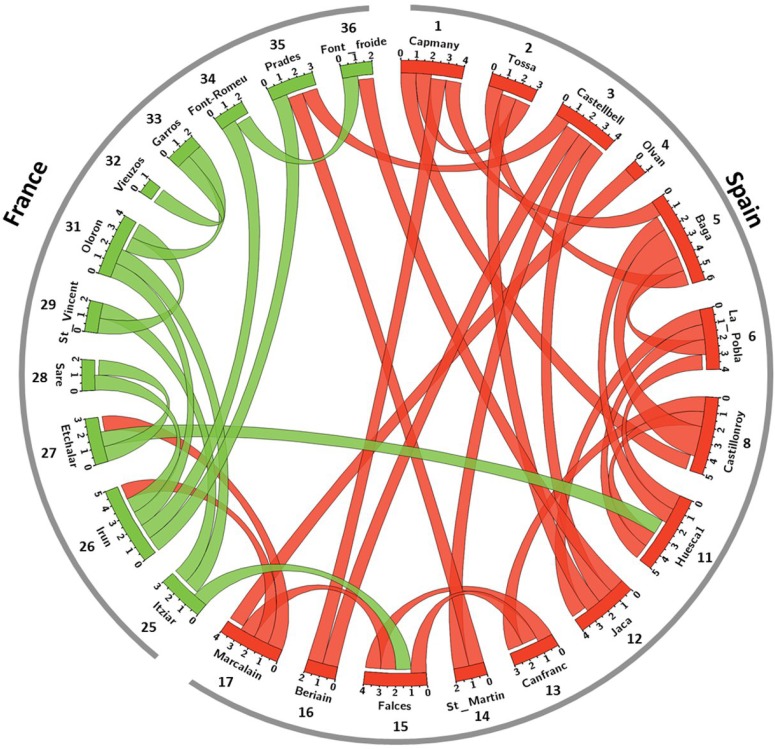
Origin of the 40 first generation migrants detected among the 26 populations tested. Populations are represented on the circle by arcs. Colors refer to the assignments of populations to the two clusters identified by STRUCTURE (red = cluster of the southern side and green = cluster of the northern side of the mountain range). Ribbons represent migrants of first generation. The attached end indicates the population of "arrival" and the detached end to the most likely population of origin of the migrant.

The rate of admixture of populations obtained with LEA analysis ranged from 6.7% to 63.3% (Fig 2). Multiple iterations gave stable results (average SD over populations = 0.5%, maximal SD = 1.1%). Admixture was higher in populations of the South hillside for transects A, B and C, suggesting an asymmetric migration of individuals, from France to Spain on the Western side of the Pyrenees. Transects A and B showed a negative relation between admixture coefficient and distance to the ridgeline. A total of 40 first generation migrants were detected among the 26 populations tested. Events of migration were mainly observed within each hillside, and particularly between neighbor populations (Fig 3). When considering the two genetic clusters defined with STRUCTURE, migration between clusters was more often observed among populations of the western hillside (Falces, Marcalain, Itziar, Irun, Etchalar). It should be noted that the origin of migrants computed by GeneClass is an estimation of the most likely population of origin which themselves may be poorly differentiated from each other within hillsides. Thus, these results show a picture of general patterns of migration within and between clusters rather than an accurate prediction of origin of migrants. Few first generation migrants were detected between populations separated by high elevations (Prades-Castellbell, Jaca-Font froide) and we did not detect a specific signature of migration along road axes in the highest parts of the mountain ranges.

### Resident populations and potential spread of PWN-infested populations

**Fig 4:**
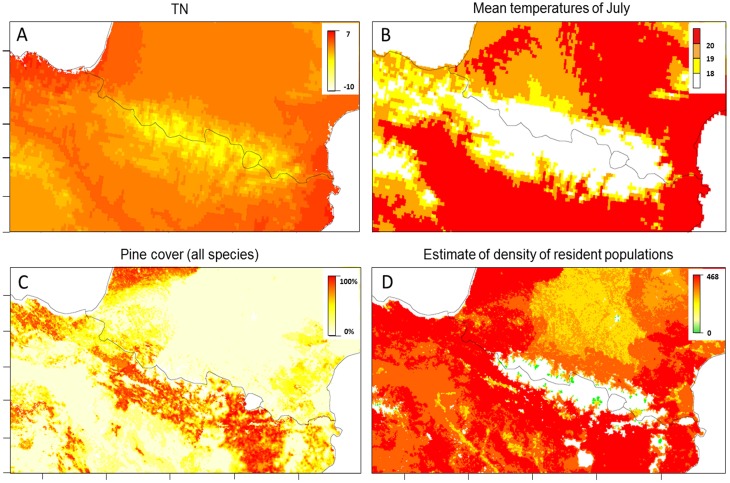
Temperatures, pine cover and resident populations in the Pyrenees. A) Mean of minimum temperature (°C) in winter (December, January and February; called TN) over 1950–2000. B) Mean temperatures (°C) in July over 1950–2000. C) Pine cover (%). D) Projection of the distribution and density of resident populations of *M*. *galloprovincialis* over the Pyrenean chain. Maximal elevation threshold for the beetle survival set to 1590 m for this representation. Scale refers to the population density (number of individuals per km^2^).

**Fig 5:**
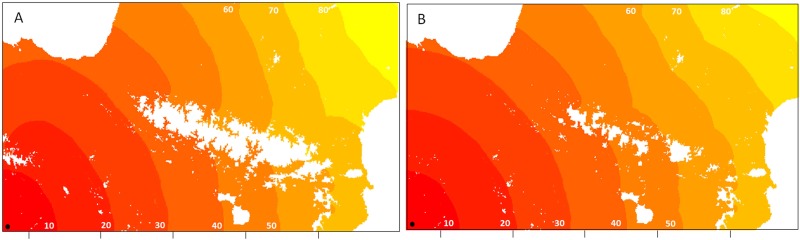
Simulations resulting from the spread model. Figures show the potential spread of infested populations of *M*. *galloprovincialis* over the Pyrenees based on pine density and limits of survival of this species. A) Simulated spread when considering a maximal elevation of 1590 m for beetle survival. B) Simulated spread when considering a minimal temperature (TN) of -7°C for beetle survival. Simulations started from Spain (black circle) and were run until complete spread over the area. Each color panel represents the area colonized by the PWN in 10 years.

**Fig 6:**
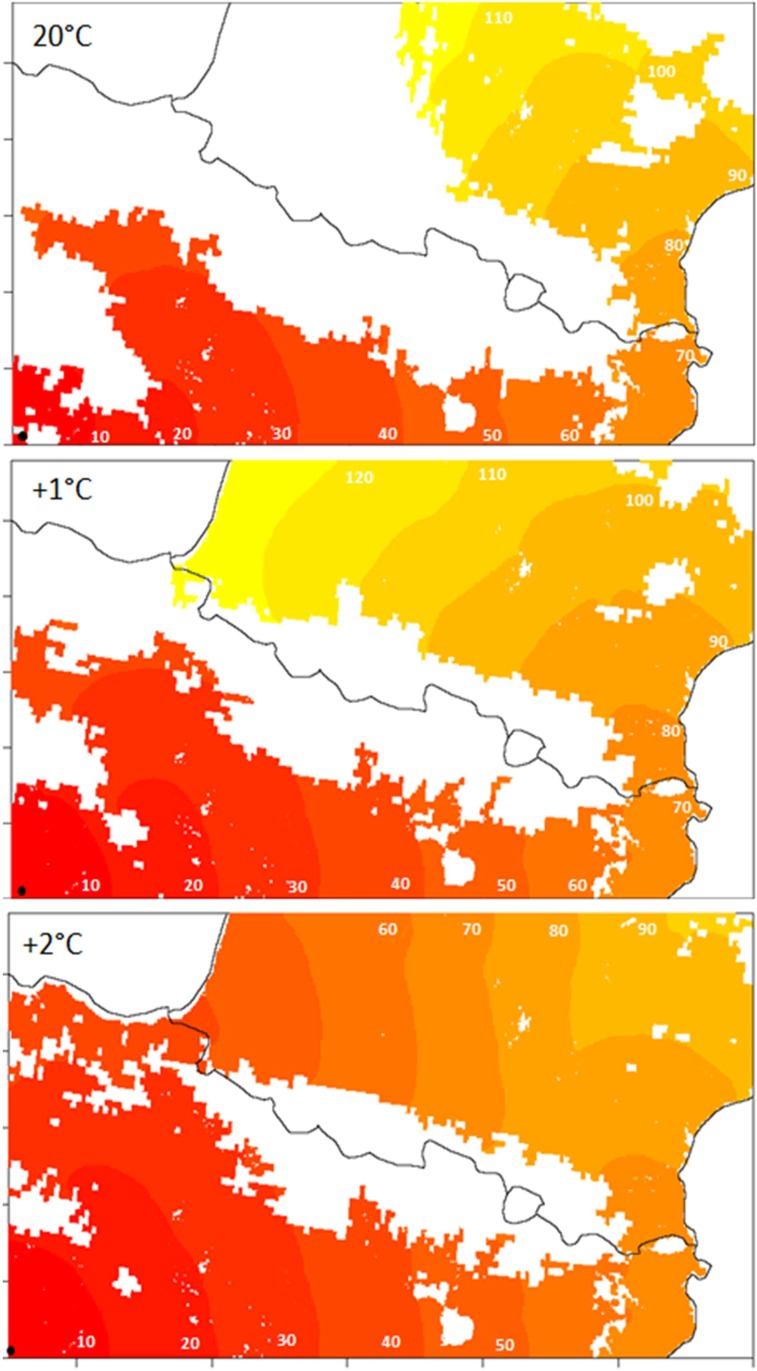
Simulated spread of PWN-infested populations assuming no spread of PWD infested beetles where the disease does not express. Maps represent the population density of infested populations for various temperature thresholds for PWD expression (mean July temperatures of 20°C and + 1°C and +2°C of climate warming). Simulations started from Spain (black circle) and were run until complete spread over the area. Each color panel represents the area colonized by the PWN in 10 years.

Based on the carrying capacity (*K*_0_) estimated across the study area, we found that *M*. *galloprovincialis* is expected to be widely distributed over the Pyrenees (Fig 4D). The highest *K*_0_ was found along the Mediterranean and the Atlantic coasts, and on the Spanish side of the Pyrenees. Conversely, low *K*_0_ was observed in an area located away from the coast on the French Pyrenean side. Based on trapping, we found an average carrying capacity of *K*_0_ = 0.027 insect / tree (S2 Table) and a ratio between *K*_i_ and *K*_0_ of *K*_i_≈4 *K*_0_ (See S3 Table for details). Results of simulations are represented by areas colonized by intervals of 10 successive generations (Figs 5 and 6). When considering no thresholds of temperature and elevation, model simulations of the spread of infested populations of *M*. *galloprovincialis* showed a wave of colonization which expanded over all suitable habitats in Spain and France.

### Thresholds of temperature and elevation

The temperature distribution over the 353 sampling sites where *M*. *galloprovincialis* was recorded in Europe showed that this species can be found over a large range of TN temperatures (S2 Fig). The species was sampled in areas with a TN ranging from -10.0 to + 9.8°C; however, 50% of sampling sites were between −2.0 and + 2.0°C. Only 6% of the sites were below -5.0°C and 2% below -7.0°C. Thus, the thresholds of -5.0 and -7.0°C for beetle survival tested seem consistent with literature and trapping records, as very few insects were recorded below these temperatures. In addition, the lowest TN where *M*. *galloprovincialis* was collected was -7°C (Col de la Pierre St Martin, transect B, Table 1). Population density in this site was very low (only 10 specimens collected over the 3 months of trapping, against an average of 131 on the other sites, S2 Table). We thus consider that this value gives a consistent approximation of temperature threshold for beetle survival. Regarding elevation, we recorded *M*. *galloprovincialis* at a maximal elevation of 1590 m (Col de la Pierre St Martin). Given the low density encountered at this site, we also considered this elevation as the most likely threshold for beetle survival. The threshold of -7°C and 1590 m defined a discontinuous barrier of 1456 km^2^ in length and a continuous barrier of 9156 km^2^ respectively along the Pyrenean chain (Fig 5). These barriers did not stop the simulated spread of infested populations and moderately affected the spread rate, due to the presence of corridors of suitable habitats located on each edge of the Pyrenean chain bounding the Atlantic and Mediterranean coasts.

Over all the simulations, none of the tested thresholds of temperature and elevation were predicted to completely stop the spread of infested vector populations across the Pyrenees. However, these thresholds affected the number of generations (i.e., years) needed to reach the opposite end of each transect (Table 2). The spread rate was not affected by changes in minimal temperature and maximal elevation for beetle survival along the most western transect (A) where elevation is lower (maximal elevation of approximately 800 m along the transect A versus 2200, 2800 and 2300 m for transects B, C and D respectively). This dynamic was moderately affected along the most Eastern transect (D) (5% and 7% of variation among the altitudes and temperature thresholds tested). The effect of changes in temperature and elevation was higher along transects B and C, located across the most elevated part of the ridgeline (21 and 19% for altitudes and 50 and 60% for temperatures).

**Table 2:** Simulations of the temporal dynamic of spread of the PWN-infested populations of *M*. *galloprovincialis* along the four transects (A, B, C & D).

**Max. alt. (m)**	1200	1500	1580	1700	2000	%
**A**	23	23	23	23	23	0
**B**	24	21	20	20	19	21
**C**	52	44	43	41	36	30.8
**D**	29	29	28	28	28	3.4
**Min. temp. (°C)**	0	-2	-5	-7	-10	%
**A**	23	23	23	23	23	0
**B**	38	23	20	19	19	50
**C**	64	51	39	26	25	60
**D**	29	28	27	27	27	6.9

Values represents the number of generations required to spread from the Spanish extremity to the French extremity of the transects. Max. alt. and Min. temp. represent respectively the maximal elevation and minimum temperatures (TN) for beetle survival tested. Percentages refer to the extent of change in number of generations between the minimum and maximum temperature and altitude tested.

When testing the temperature threshold for PWD expression (20°C), and potential effect of climate warming of +1°C and +2°C, an unfavorable area of 88,281, 58,402 and 42,173 km^2^ were determined over the Pyrenees (Fig 4B). Potential spread of infested populations was very different under warming scenarios (Fig 6). The temperature threshold of 20°C created a wide unfavorable area for spread of PWN on the western site of the Pyrenees, but 1°C and 2°C increases in temperature reduced considerably this area. All the scenarios tested predicted a corridor suitable for PWD expression and spread of infested *M*. *galloprovincialis* along the Mediterranean hillside of the Pyrenean chain.

## Discussion

New phoretic combinations resulting from introductions of alien species have been documented for several insect-pathogen relationships. They involve either an endemic pathogen spread by an introduced insect [65, 66], or conversely an exotic pathogen spread by a native insect [67]. Phoretic associations leading to major environmental disturbances have been well studied [68, 69]. However, few studies have addressed the question of their dispersal in a heterogeneous landscape.

In Europe, *M*. *galloprovincialis* is already associated with the endemic, non-pathogenic species *Bursaphelenchus mucronatus* [70]. A novel phoretic association with *B*. *xylophilus* would improve the fitness of *M*. *galloprovincialis* by providing temporally more declining trees for development. To assess the potential spread of the PWN, both partners of this nematode-beetle association and their synergic effects must be considered. In this study, we combined population genetic and modeling approaches to explore the effects of the Pyrenean chain on the spread of PWN-infested populations of *M*. *galloprovincialis*. We show that the Pyrenees represents a partial barrier to northward/southward migrations of beetle populations and we identified potential corridors for the natural spread of the PWN. This integrative study provides the first analysis of the potential dispersal of the PWN-vector association at local geographic scale in Europe and highlights priority zones for future pest management programs along the Pyrenean barrier.

### Genetic structure and differentiation of populations

Our population genetic analyses provide evidence that the Pyrenean chain has acted and continues to act as a barrier to dispersal of *M*. *galloprovincialis*. We found population differentiation (pairwise *F*_st_) systematically higher and more significant between populations from each side of the mountain ridge than within hillsides. In addition, the clustering method clearly identified two genetic lineages distinct to the northern and southern sides respectively, forming a zone of separation along the ridgeline of the mountain range. This result is in agreement with the genetic differentiation observed for *M*. *alternatus* across a mountain range in Japan [71]. From an evolutionary perspective, such a genetic structure suggests the presence of a secondary contact zone of two differentiated lineages of *M*. *galloprovincialis* along the Pyrenees, consistent with the phylogeographic patterns documented for several native species in Europe including trees [72, 73], vertebrates [74] and insects [75]. In our case, the area studied should be expanded to a wider phylogeographic context to validate this scenario.

The overall genetic differentiation of the populations was low to moderate but consistent with intraspecific population differentiation encountered at local scale for flying beetles [71, 76, 77, 78]. We found a significant isolation by distance correlation in the studied area, which is compatible with results found for *M*. *alternatus* in Japan [71]. The low genetic differentiation within hillsides suggests intensive gene flow and subsequently rather high dispersal abilities for *M*. *galloprovincialis*. This corroborates the important dispersal distances estimated in laboratory conditions [22] and in the field [23] for this species. This result is also consistent with observation made on the congeneric species *M*. *alternatus* for which low population differentiation was found between populations in lowland valleys [71]. Our results tend to show that *M*. *galloprovincialis* does not encounter strong barriers to dispersal in lowland valleys to the North and South of the Pyrenees. We found no signal of population differentiation within the altitudinal gradients of hillsides. Altitudinal gradients of genetic differentiation have been encountered in the salamander *Ambystoma macrodactylym*, based on microsatellite markers [79] and in the alpine butterfly *Lycaena tityrus*, based on genes under selection [80]. Our observation suggest no clear differentiation between populations in high and low elevation areas, and subsequently no differentiation between insects feeding on thermophile Mediterranean pines (*P*. *halepensis*, *P*. *pinaster*) and the ones feeding on pines encountered at altitude (*P*. *sylvestris*, *P*. *uncinata*). These results are consistent with feeding experiments showing that this species may develop on almost all pine species encountered in this zone [57]. This analysis did not provide evidence to suggest that changes of pine species along hillsides of the Pyrenees drive divergence and gene flow in this species. Absence of differentiated lineage in an elevated area can be a sign of recent expansion of the species in altitude. We did not observe recent bottlenecks among populations, indicating that the genetic patterns observed are better explained by long term processes than from recent spatial expansion in these areas.

Genetically differentiated lineages of *M*. *galloprovincialis* associated with elevation or specific host pines are important to consider as they may show different responses once in contact with the PWN. The absence of structured populations with altitude did not support the existence of the former altitudinal subspecies *M*. *galloprovincialis pistor* (Germar, 1818) [81] characterized by darker teguments. For the Pyrenean populations analyzed in this study, our results are in agreement with morphological analysis [82] suggesting no evidence of a genetic base for the validity of this former subspecies. As a result, management does not need to take into account multiple lineages for *M*. *galloprovincialis* in the Pyrenean area.

### Corridors and barriers to dispersal of *M*. *galloprovincialis*

The presence of two genetically differentiated lineages on each hillside enables detection of migration and long-distance dispersal events across the mountain range. We found higher gene flow on the western and eastern sides of the mountain range, and especially along the most western transect (A). This is probably due to the moderate elevation and the presence of a continuous pine tree cover encountered in this area. Bayesian assignment based on individuals also argues for a higher admixture rate among populations of transect A. The western and eastern extremities of the Pyrenean chain are known corridors for lineage expansion of several species during recent post-glacial re-colonization events [83, 84, 85]. Similarly, distribution of mitochondrial haplotypes of the pine processionary moth (*Thaumetopoea pityocampa*) suggests a recolonisation route of this species from Spain to France through the western side of the mountain range [86]. The pine processionary moth develops on a large range of pines species. Thus this indicates that the western side of the mountain range was a continuous corridor of pines at least temporarily over the current interglacial period (Holocene, see Bucci et al. 2007 [87] for distribution of genetic clusters of *P*. *pinaster* in this area). In the case of *M*. *galloprovincialis*, however, although migration was detected in this area, our results show the presence of a separation zone, as observed in the grasshopper *Chorthippus parallellus* [75]. The maintaining of this zone despite favorable habitats and gene flow is unexpected and may be related to the effect of isolation by monopolization or by isolation by local adaptation of the two lineages to local environmental conditions [88].

Long-distance dispersal events, caused by human transportation are critical parameters, because they can significantly accelerate the spread of PWN [37]. Such transportations may disperse beetles bypassing unfavorable areas (i.e. high elevations) that would naturally stop the migration. The Pyrenees is the only land connection between the Iberian Peninsula and the rest of Europe, and the road traffic along the border between Spain and France is intense. Around 120,000 vehicles cross the border each day along 27 axes. The majority of traffic (67%) occurs in the coastal zones [89]. The traffic in elevated areas is very low and scattered, except on the Eastern side of the mountain range (transect D), in Andorra and Bourg-Madame [89]. This traffic may contribute significantly to long-distance dispersal of the beetle and we searched for genetic signatures of such events. However, we did not find evidence of long-distance dispersal along specific road axes in the high mountains. This suggests that human transportation of *M*. *galloprovincialis* along these axes is absent or not frequent enough to be detected using the methods employed in this study. Conversely, we observed higher migration events in the western and eastern hillsides of the Pyrenees where most of the traffic is concentrated. Estimating the relative importance of human mediated versus natural dispersal as determinants of genetic patterns is challenging. In this study, we found that individuals disperse more from North to South of the ridgeline in the western hillside. This situation contradicts the patterns of northward natural expansion of Spanish populations observed for other species [85, 86, 90] in this area. This case may be related to intensive human mediated dispersal, caused by the transport of a massive amount of pine logs to Spain after windstorms that occurred in Southwestern France [91]. Such hypothesis should be considered with care given the lack of data regarding the temporal scale at which the genetic patterns observed were formed.

Estimation of gene flow assessed in this study provides an overview of effective dispersal of *M*. *galloprovincialis*. It informs about the distance between the place of birth and reproduction of individuals over generations. This provides a general picture of migration patterns of the beetle in the Pyrenees, but does not necessary relate to effective dispersal of PWN which can be transmitted without reproduction of the beetle through maturation feeding. The modeling approach allowed us to assess potential expansion of the PWN including an estimate of effective dispersal of the nematode.

### Estimated density of resident populations

The resident population density derived from the pine tree density suggests that *M*. *galloprovincialis* is present in all areas within the mountain range, at least at low population density. This widespread presence indicates that suitable host plants for *M*. *galloprovincialis* are universally available in this area and that habitat connectivity is important. Such conclusions are supported by trapping observations, showing that this species is present in almost all sites sampled including the ones with very low pine density, and where trees are present as very scattered pine stands (Oloron, Vieuzos). In addition, over the study area, all pines species were found to host *M*. *galloprovincialis*. We even trapped specimens in an isolated plot of *P*. *pinea* in Spain (Huesca 1) which is described as unsuitable for its larval development [57]. These specimens may either be locally adapted to this host, or may come from distant location and other pine species, pulled into the pheromone traps. Thus, according to our observations and simulations and the widespread distribution of suitable pine tree host species, the distribution of the vector beetle is not a limiting factor to PWN spread in most of the studied area.

### Potential spread of PWN across the Pyrenean chain

The model used in this study to simulate the potential spread of infested populations of *M*. *galloprovincialis* was refined from a previous model of PWN spread [37, 38]. Our model took into account the chronological steps in recruitment of resident populations and the increase in population density due to tree mortality in PWN-infested zones. This allowed us to consider more accurately interactions between the two species in the simulations of dispersal of PWN infested populations. From sensitivity analysis of the model, *D* (diffusion coefficient) appears to have the greatest effect on results compared to the growth rate (*Ɛ*) and the carrying capacity (*K*_0_). Simulated spread supports the genetic structure and migration patterns observed for this species. Thresholds of temperature and elevation tested always defined at least a moderate barrier to dispersal at high elevation within the mountain range but identified corridors of dispersal on the Eastern and Western sides of the ridge line. Thus, according to these results based on biological and physical parameters of survival of *M*. *galloprovincialis*, the Pyrenean chain constitutes only a partial barrier for the spread of PWN-infested populations.

When considering the temperature threshold for PWD expression (mean July temperatures above 20°C), high elevations and the western side of the Pyrenean chain formed potential wider barriers to the spread of PWN-infested populations. This result suggests a higher potential of temperatures threshold for PWD expression to slow the spread of PWN across the mountain range compared with thresholds of beetle survival. This is in agreement with results found in China, where simulations predicted an effect of elevation on the spread of PWD [37, 92], but in contrast to the very high mountains in China, the Pyrenees (lower elevation) would have a weaker effect in case of climate warming. To confirm these results, there is a need to refine precisely this threshold in Europe and to quantify the spread of the PWN in areas where the PWD does not express. In addition, it should be noted that temperatures used in this study are based on records from recent decades (1950–2000) and that given the current climate change, the scenarios considering temperature increase of +1 and +2°C are probably more realistic. Current development of models for PWD expression in Europe will help to refine these predictions [93].

Future climate change and particularly temperature rise may affect the spread of the PWN by providing new areas suitable for PWD development. In this study, a moderate temperature rise of +1 and +2°C reduced considerably the potentially unsuitable area for PWD expression and connected suitable area on the western side of the mountain range. This indicates that even a moderate climate change may affect significantly the range expansion of PWD by reducing the extent of potential barriers as pointed by Roques et al. 2014 [92]. Temperature increase may also widen the range of habitat of *M*. *galloprovincialis* in elevated areas. Due to uncertainties on the temperatures of survival threshold of this species, it was not possible to formally test the effect of climate change on spread of infested beetles based only on beetle survival.

Our simulations are based on existing knowledge on *M*. *galloprovincialis*, the PWN and their interactions. However, several parameters such as PWN transmission efficiency, the role of a sister species found in elevated areas in PWN transmission (*M*. *sutor*), the dispersal behavior of infested beetles and environmental factors affecting PWD expression are still lacking accurate data. Future studies will have to address these questions to refine the spread model and better predict invasion routes of this pest in Europe. Long-distance jumps resulting from human transportation is an important parameter to be considered in the potential spread of PWN. Although spread models taking into account human mediated dispersal have been applied to Europe, long-distance dispersal of PWN in Europe is still poorly understood and lacks observed data [38]. Lastly, early detection and eradication of new outbreaks of PWN-infested populations may strongly affect PWN spread. Clear-cut belts may also create artificial barriers to the spread of this pest. These control measures and the efficiency of their application are obviously critical parameters to consider in the potential range expansion of the PWN in Europe.

## Conclusion

This study aimed at evaluating the effect of an altitudinal barrier on the natural spread of PWN infested populations of *M*. *galloprovincialis*. Based on gene flow estimates of the beetle and on the simulations resulting from a refined spread model, we showed that the Pyrenean chain constitutes a partial barrier to the natural spread of this alien from Spain to the rest of Europe. Our results suggest that the western and eastern parts of this mountain range will not constitute effective barriers to prevent the spread from Spain to France, although the western side appears less favorable to PWD expression under current climate conditions. These areas should be considered as priority zones for pest monitoring and management programs. Barriers to dispersal of *M*. *galloprovincialis* and spread of the PWN highlighted here are potentially relevant to others mountainous systems in Europe (Alps, Carpathians) exhibiting similar biological and physiological constraints.

## Supporting Information

S1 Fig Location of sampling sites and road axes in the Pyrenees. (EPS)

S2 Fig Distribution of the mean of minimal temperature in winter over 1950–2000 (TN) for 353 sampling sites of *M*. *galloprovincialis* in Europe. (EPS)

S1 Table Unbiased pairwise *F*_st_ estimates of the 26 populations considered. (XLSX)

S2 Table Estimation of the carrying capacity (*K*). The number of insects per tree was evaluated based on density of individuals trapped and the number of trees in the area surrounding the trap. For this calculation, we consider only localities for which traps were set during the same duration (4 months). Traps were set in dense and large pine plots approximating a cover of 100%. Assuming that 100% pine cover represents a number of 156000 trees/km^2^, we found a number of 4898 trees in a surface of radius of 100 m around the sampled sites (estimated area of attraction of the traps). We then report the number of individuals trapped to the number of trees estimated in the surface influenced by the traps. We found an average value of 0.027 insect per tree. Sensitivity analysis (see appendix 3) shows that variation of *K* has very low impact on model results. Subsequently, uncertainties on estimation of this parameter are not likely to affect strongly model results.
(XLSX)

S3 Table Estimating the ratio between *K*_0_ and *K*_i_. Traps (Multifunnel and specific attractant volatile) were set in PWN free and PWN-infested pine stands (*Pinus pinaster*) in Portugal and Spain during July 2013. We counted specimens caught during 2 weeks of trapping. We estimated the population density as on average four times higher in PWN-infested areas than in PWN free areas (*K*_i_ ≈ 4*K*_0_).
(XLSX)

S4 Table Sensitivity analysis. The model sensitivity to the main parameters (*D*, *ε* and *K*_*i*_) was assessed by varying the parameters’ value by +/- 10% from the baseline values previously given. The effect of these changes was quantified in terms of infested area (km^2^) and percentage of change compared to the baseline settings. Simulations were done from *t* = 1 to 20 years, and from 3 different starting points (P1: 41.68° N, -2.94° E; P2: 41.68° N, 0.81° E and P3: 41.68° N, 2.73° E) to assess a possible effect of the starting point on parameter’s sensitivity. Among the 3 parameters tested, variations in diffusion coefficient (*D*) involved the largest changes in area colonized. Others parameters were affected by less than 2%.
(XLSX)

S5 Table Microsatellite dataset. (TXT)
